# Enhanced production and extraction of phenolic compounds from wheat by solid-state fermentation with *Rhizopus oryzae* RCK2012

**DOI:** 10.1016/j.btre.2014.09.006

**Published:** 2014-09-20

**Authors:** Tapati Bhanja Dey, Ramesh Chander Kuhad

**Affiliations:** Lignocellulose Biotechnology Laboratory, Department of Microbiology, University of Delhi South Campus, Benito Juarez Road, New Delhi 110021, India

**Keywords:** Wheat, Solid-state fermentation, Phenolics, Extraction, Free radical, Antioxidant

## Abstract

Antioxidant phenolic compounds (PCs) are gaining popularity day by day for their health promoting properties. Wheat is a very good source of natural antioxidant PCs. In the present study, extraction of PCs was improved by solid-state fermentation (SSF) of wheat by *Rhizopus oryzae* RCK2012 which helped to release the bound compounds from matrix. Different extraction conditions such as solvent composition (water, methanol, 70% methanol, ethanol, 70% ethanol, acetone and 70% acetone), extraction temperature (30–60 °C), extraction time (15–90 min) and solid-to-solvent ratio (1:2.5 to 1:20, w/v) have been optimized for the extraction of PCs from *R. oryzae* fermented wheat. Maximum PCs were extracted by water at 40 °C within 45 min with solid-to-solvent ratio of 1:15 (w/v). Compositional analysis of PCs was carried out by UPLC and TLC. Improved ABTS^•+^ [2,2′-azinobis(3-ethylbenzothiazoline-6-sulfonic acid)] and DPPH (2,2-diphenyl-1-picrylhydrazyl), hydroxyl radical and hydrogen peroxide scavenging capacities, ferric reducing property and *in vivo* antioxidant capacity using *Saccharomyces cerevisiae* were observed in case of freeze-dried water extract of fermented wheat as compared to unfermented sample. Hence, SSF could be a promising technology to enhance the production and extraction of phenolic compounds for the design of different functional foods and for the specific use as nutraceuticals.

## Highlights

• SSF was done for the enhanced production and extraction of phenolics antioxidants from wheat.
• A newly isolated *Rhizopus oryzae* RCK2012 was used in SSF.
• Different conditions were optimized for the extraction of phenolics from fermented wheat.
• Compositional analysis of released phenolics was carried out by UPLC and TLC.

## 1 Introduction

“Oxidative stress” may occur due to an imbalance between oxidants and antioxidative defense system of human body. Under this condition excessively produced reactive oxygen species (ROS) and free radicals damage different biological molecules, such as DNA, proteins, lipids as well as carbohydrates with significant molecular and physiological damages of cells leading to numerous diseased conditions [15]. Plant-derived different antioxidant molecules with their reducing, free radical scavenging and metal chelating properties can reduce oxidative stress keeping equilibrium between oxidants and antioxidants in human body [2].

Phenolic compounds are mostly studied diversified group of phytochemicals synthesized from phenylalanine and tyrosine by the enzymatic action of l-phenyloalanine ammonia-lyase, PAL (EC 4.3.1.5) in secondary metabolic pathway of plants during normal developmental stage or in stressed conditions by ecological and physiological pressures including infection by pathogen or insect, wounding and UV-radiation etc. [24], [33]. Over the last few decades, they have become popular for their potential application in the prevention of various chronic diseases, viz. cardiovascular disease, cancer, osteoporosis, diabetes mellitus, and neurodegenerative diseases etc. They protect cells by their antioxidant properties [21]. Over the last few years, various natural sources of different antioxidant phenolic compounds have been explored including fruits, vegetables, wines, coffee, tea, pulses and cereals in order to restrict the use of health hazard synthetic antioxidants like butylated hydroxyanisole (BHA), butylated hydroxytoluene (BHT) and tertiary butyl hydroquinone (TBHQ), in different food products.

Different conventional solvent extraction (liquid–liquid/solid–liquid) strategies have been employed for the extraction of phenolics from plant materials like Soxhlet extraction, maceration, microwave-assisted extraction, ultrasound-assisted extraction, high hydrostatic pressure extraction and supercritical fluid extraction etc. [18]. Whole grain wheat is a very good source of bioactive phenolic compounds. Extraction and isolation of phenolic components of wheat are difficult because those compounds are present as insoluble bound form conjugates with sugars, fatty acids or amino acids. According to Adom and Liu [1] about 90% phenolics are present as bound form in wheat. Hence, without acid/base hydrolysis, extraction of most of the insoluble bound phenolics is difficult by only organic solvents. Extraction of natural phenolics by enzymatic treatment is a useful technique. Several microorganisms are known to produce a variety of enzymes in high titer values preferably under solid state fermentation (SSF) process. Recently, SSF has gained a considerable attention for the production and extraction of antioxidant phenolics from plant materials, mainly pulses and cereals [21]. In this process, different carbohydrases like cellulases, β-glucosidase, xylanase, pectinases, β-xylosidase, β-galactosidase, α-amylases and esterase etc., produced by the microorganisms can release the bound phenolics into soluble form [2].

In the present report, production and extraction of phenolics were improved through SSF of wheat grains by *Rhizopus oryzae* RCK2012. A single standardized method should not be recommended for the extraction of all types of phenolic compounds. Extraction process has to be optimized depending upon the nature of the sample and purpose of the study [26]. In this study, different extraction conditions such as solvent composition, extraction temperature, solvent-to-solid ratio and extraction time have been optimized for the extraction of phenolics from *R. oryzae* RCK2012 fermented wheat grains. Furthermore, comparative studies have been carried out between fermented and unfermented wheat on the different antioxidant properties of freeze-dried water extracts. Some studies already have been carried out for the improvement of total phenolics and antioxidant properties of wheat bran [22], rice [3], maize [10], wheat [2], [4], buckwheat, wheat germ, barley and rye [11], oat [6], [7], oat, wheat, buckwheat and pearl barley [30] and rice bran [27] utilizing various food grade microorganisms. To the best of our knowledge, this is the first report on optimization of different extraction conditions of phenolic antioxidants from the *R. oryzae* fermented wheat grains.

## 2 Materials and method

### 2.1 Materials

Following chemicals were procured from Sigma–Aldrich chemicals (USA): 2,20-diphenyl-1-picryl-hydrazyl (DPPH), 2,2′-azinobis (3-ethylbenzothiazoline-6-sulfonic acid) diammonium salt (ABTS), trolox, phenolic acid standards such as gallic, protocatechuic, caffeic, 4-hydroxy benzoic acid, 4-hydroxy 3-methoxy benzoic acid, trans-cinnamic acid and ferulic acid. All other chemicals were analytical grade.

### 2.2 Organism, inoculum preparation and substrate

A new fungus was isolated locally from rotten maize and identified as *Rhizopus oryzae* RCK2012 (GenBank Accession No. JQ906263). It was cultivated and maintained on potato dextrose agar (PDA). Inoculum was prepared from 3 days old slant by suspending the fungal spores in sterile distilled water and adjusted to a concentration of 1 × 10^6^ spores/ml. One batch of commercial wheat grains were stored at room temperature and were used throughout the experiments.

### 2.3 SSF of wheat

Ten gram of whole grain wheat taken in 250 ml Erlenmeyer flasks, was mixed with 10 ml distilled water, autoclaved (121 °C, 15 min) and subsequently cooled to ambient temperature. Fungal spore suspensions (1 × 10^6^ spores/ml) were inoculated separately (at 10% inoculum ratio, w/v) onto the surface of the steamed substrates, mixed properly and incubated for 3 days at 30 °C. The unfermented wheat (control) was prepared without addition of spore suspension.

### 2.4 Optimization of phenolic compounds extraction conditions

The fermented mass was taken out of the Erlenmeyer flask after 3 days, autoclaved and dried in an oven at 60 °C for 24 h. The dried unfermented and fermented substrates were ground in an electric grinder. All samples tested were defatted by blending the ground material with hexane (1:5 w/v, 5 min, thrice) at ambient temperature. Defatted samples were air dried for 24 h and stored at −20 °C for further analysis. Defatted and air dried samples were extracted with solvents [1:10 w/v] twice at 50 °C for 60 min in water bath. After filtering through Whatman No.1 filter paper, the filtrate was used for comparative study of total phenolic content and determination of %DPPH scavenging antioxidant property.

In order to observe the effect of different temperatures for the extraction of phenolics, unfermented and fermented wheat were extracted with water, methanol, 70% methanol, ethanol, 70% ethanol, acetone and 70% acetone at different temperatures (23–60 °C) for 60 min.

Whereas, to find out the effect of alcohol concentration on extraction of total phenolic compounds, phenolic compounds were extracted from fermented wheat using different methanol and ethanol concentration, ranging from 40% (v/v) to 90% (v/v) at optimum temperature for 60 min. Moreover, effect of extraction time (15–90 min) and effect of solid-to-solvent ratio (1:2.5–1:20; w/v) were evaluated for the maximum extraction of antioxidant phenolic compounds from fermented wheat.

Water extract derived from unfermented wheat (UFW) and the newly isolated strain *Rhizopus oryzae* RCK2012 fermented wheat (ROFW) were freeze-dried and stored in sealed vials at 4 °C for further analysis.

The extraction yield was calculated by the following equation:

$$|\text{Extraction }\text{yield}\%=\frac{\text{Weight of }\text{freeze}-\text{dried}\text{extract }(\text{g})}{\text{Weight}\text{of defatted}\text{ sample}(\text{g})}|\times 100$$

### 2.5 Analytical methods

#### 2.5.1 Determination of total phenolic content (TPC)

Total phenolic content was estimated according to Emmons and Peterson [12]. Suitably diluted 0.5 ml aliquots from phenolic extracts were mixed with 0.5 ml Folin–Ciocalteu reagent. Then 1.5 ml of 20% aqueous sodium carbonate solution was added, mixed properly and incubated for 15 min at room temperature. The samples were diluted with 5 ml of distilled water and absorbance was recorded at 725 nm against a blank. The amount of total phenolic was calculated as gallic acid equivalent (GAE) from the standard calibration curve of gallic acid and expressed as mg GAE g^−1^ grain.

#### 2.5.2 DPPH (2,2-diphenyl-1-picrylhydrazyl) radical scavenging assay

The free radical scavenging activity of different fractions was measured by the DPPH radical scavenging method according to Brand-Williams et al. [5]. DPPH (Sigma–Aldrich Chemie, Steinheim, Germany) solution of 0.1 mM concentration in methanol was added to 0.5 ml of properly diluted phenolic extracts. The change in absorbance at 515 nm was measured after 30 min of incubation. The DPPH radical-scavenging activity of phenolic extract was calculated according to the following equation.

$$\text{\% of }\text{DPPH}\text{ radical }\text{scavenging activity}=\left[\frac{\left(\text{AbC}-\text{AbS}\right)}{\text{AbC}}\right]\times 100$$

where, AbC was the absorbance of the control and AbS was the absorbance in the presence of the test compound.

A standard curve was prepared by using different concentrations of Trolox. The DPPH scavenging activities of phenolic extracts were expressed as μmol Trolox equivalent (TE) g^−1^ grain.

#### 2.5.3 ABTS radical cation scavenging assay

It was determined following the improved ABTS decolorization assay method of Re et al. [24]. ABTS^•+^ was generated by oxidation of ABTS with potassium persulphate. One milliliter of ABTS^•+^ solution was mixed with 10 μl of water extract and the decrease of absorbance was measured after a reaction time of 1 min. Similar to DPPH scavenging activity, ABTS^•+^ scavenging property was expressed as μmol TE g^−1^ grain.

#### 2.5.4 FRAP (ferric reducing antioxidant property) assay

It was estimated by the method of [29] with some modifications. The freeze-dried water extract (100 μl) of UFW and ROFW at different concentrations (2.5–10 mg/ml) was mixed with 1.5 ml of FRAP reagent (10 parts of 300 mM sodium acetate buffer at pH 3.6, 1 part of 10 mM TPTZ solution and 1 part of 20 mM FeCl_3_, 6H_2_O solution) followed by incubation at 37 °C in a water bath for 30 min. Then the increase in absorbance was measured at 593 nm. FRAP values were expressed in terms of mM ascorbic acid equivalent (AAE)/ml using l-ascorbic acid as standard.

#### 2.5.5 Hydroxyl radical scavenging assay

The scavenging capacity for hydroxyl radical was estimated following the method of Halliwell et al. [16]. The reaction mixture contained 0.1 ml of 1 mM EDTA, 0.01 ml of 10 mM FeCl_3_, 0.1 ml of 10 mM H_2_O_2_, 0.36 ml of 10 mM deoxyribose, 1.0 ml of different concentrations (0.01–0.1 mg/ml) of freeze-dried water extract of UFW, or ROFW, 0.33 ml of phosphate buffer (50 mM, pH 7.4) and 0.1 ml of 1 mM ascorbic acid in sequence. After incubation at 37 °C for 1 h, about 1.0 ml of the incubated mixture was mixed with 1.0 ml of 10% TCA and 1.0 ml of 0.5% TBA to develop the pink color and the absorbance was recorded at 532 nm.

$$\%\text{Hydroxyl radical }\text{scavenging activity}=\left[\frac{\text{AbC}-\text{AbS}}{\text{AbC}}\right]\times 100$$

where, AbC = absorbance of the control and AbS = absorbance in the presence of test sample.

#### 2.5.6 Hydrogen peroxide scavenging activity

Method of Ruch et al. [25] was used for the estimation of H_2_O_2_ scavenging property. The freeze-dried water extract (0.4 ml) of UFW and ROFW at different concentrations (0.01–0.05 mg/ml) was mixed with 0.6 ml of 40 mM H_2_O_2_ prepared in 0.1 M phosphate buffer (pH 7.4). After 10 min incubation the absorbance was noted at 230 nm. A separate blank sample was used for background subtraction for each concentration.

$$\%{\text{H}}_{2}{\text{O}}_{2}\text{scavenging}\text{activity}=[1-\frac{\text{AbS}}{\text{AbC}}]\times 100$$

Where, AbC = absorbance of the control and AbS = absorbance in the presence of test sample.

### 2.6 *In vivo* antioxidant capacity using *Saccharomyces cerevisieae*

*Saccharomyces cerevisieae* was cultured for 24 h in a 50 ml volume of MGYP media (Malt extract; 3 g/l, Glucose; 20 g/l; Yeast extract; 3 g/l; Peptone; 5 g/l) by inoculating a single colony. This primary culture was inoculated [1%] in five culture tubes containing 5 ml of MGYP media and incubated at 30 °C in shaking condition at 150 rpm. After 6 h of incubation, 100 μl of ascorbic acid (10 mg/ml) as positive control and two freeze-dried phenolic extracts (10 mg/ml), namely, UFW and ROFW were added separately in each tube. Then 10 μl of hydrogen peroxide (H_2_O_2_) as oxidant was added in each tube. Growth of the yeast culture was monitored taking absorbance at 600 nm at the end of 20 h. The effect of phenolic extracts in presence of oxidants on the net growth of yeast cells was determined according to the following equation.

$$A_{\text{yeast }\text{growth}}=\frac{A_{\text{test sample}}-A_{\text{control}}}{A_{\text{contro}l}}\times 100$$

Where *A*_yeastgrowth_ = net growth of H_2_O_2_ induced yeast cells after treatment with phenolic extracts, *A*_control_ = absorbance of yeast cells in presence of H_2_O_2_, *A*_testsample_ = absorbance of yeast cells in presence of H_2_O_2_ and phenolic extracts.

### 2.7 Thin layer chromatography (TLC) and ultra-performance liquid chromatography (UPLC)

Water extracts (50 ml) of unfermented and fermented wheat were extracted with ethyl acetate [1:1; v/v] for 30 min in a separating funnel. The ethyl acetate fractions were evaporated to dryness and reconstituted in methanol. Now the phenolic extract was filtered through 0.45 μm Supor^®^-450 membrane disc filter (Pall Gelman Laboratory, USA) and then thin layer chromatography (TLC) of PCs was performed on silica gel plates using mobile phase chloroform:methanol:formic acid [85:15:1; v/v/v] and visualized under short wave (254 nm) and long wave UV light (365 nm). Same samples (2 μl) were analyzed by an ultra-performance liquid chromatography (Waters, Milford, USA). The separation of phenolics was performed on a BEH 300C-18 column (2.1 mm × 50 mm, 1.7 μm). The column temperature, total run time and flow rate were set at 30 °C, 5 min and 0.6 ml/min, respectively. Two mobile phases consisted of water containing 0.1% TFA (solvent A) and acetonitrile containing 0.1% TFA (solvent B) were used and gradient elution was carried out using the following program: 95% A to 90% A in 1 min, 90% A to 85% A in 1 min, 85% A to 75% A in 1 min, 75% A to 40% A in 1 min, 40% A to 0% A in 0.2 min, 0% A to 0% A in 0.6 min and 0% A to 95% A in 0.2 min. The peaks were identified by congruent retention times and UV spectra (280 nm) and compared with standards and quantified based on their peak’s area.

### 2.8 Statistical analysis

The mean values and the standard deviations were calculated from the data obtained from experiments in triplicates. The data were analyzed by one-way analysis of variance (ANOVA).

## 3 Results and discussion

### 3.1 Effect of extraction conditions

#### 3.1.1 Effect of different temperatures for the extraction of phenolics with different solvents

It is well known from literature data that extraction conditions and characteristics of the sample can affect the efficiency of the extraction, independently or interactively. The solvent and the temperature are the process parameters that usually have the greatest impact on the efficiency of extraction of bioactive compounds from the plant material. In general, alcohol/water solutions exert a better influence on the extractability of phenolic compounds in comparison to the mono-component solvents. It is noted that a solvent system for extraction is selected according to the purpose of extraction such as preparation or analysis, the nature of interested components, the physicochemical properties of the matrix, the availability of reagents and equipment, cost and safety concerns [31].

The amount of extracted phenolic compounds obtained in this study by different solvents at different temperatures (30–60 °C) is presented in Table 1. In case of unfermented wheat (control), maximum TPC was attained in 70% acetone extract at 60 °C (1.1 mg GAE g^−1^ grain). Whereas, in case of *R. oryzae* fermented wheat, maximum TPC (6.78 mg GAE g^−1^ grain) was obtained in water extract at 40 °C. A comparable amount of TPC was extracted by the same solvent (6.7 mg GAE g^−1^ grain) at 50 °C. Almost same amount of phenolics were released from fermented wheat by 70% methanol (5.92 mg GAE g^−1^ grain) at 40 °C and 70% acetone at 50 °C (5.89 mg GAE g^−1^ grain) and 60 °C (5.89 mg GAE g^−1^ grain). Similarly, there was no significant difference of TPC of fermented wheat extracted by 70% ethanol at 30 °C (6.4 mg GAE g^−1^ grain), 40 °C (6.19 mg GAE g^−1^ grain) and 50 °C (5.92 mg GAE g^−1^ grain). If we consider the water soluble phenolics, it was clearly observed that SSF by *R. oryzae* RCK2012 increased the TPC of wheat by ∼11 fold at 40 °C. Recently, Schmidt et al. [27] observed only 2 fold increment of TPC in rice bran after SSF by *R. oryzae.*

**Table 1:** TPC and DPPH scavenging property of phenolics extracted at different temperatures with different solvents.

Solvents	TPC (mg GAE g^−1^ grain)							
	DPPH• scavenging property (μmol TE g^−1^ grain)							
	30 °C		40 °C		50 °C		60 °C	
	Control	Fermented	Control	Fermented	Control	Fermented	Control	Fermented
Water	0.44 ± 0.12	5.46 ± 0.20	0.62 ± 0.02	6.78 ± 0.15	0.77 ± 0.03	6.70 ± 0.30	0.98 ± 0.06	5.82 ± 0.22
	1.24 ± 0.05	4.95± 0.26	1.29 ± 0.10	8.85 ± 0.05	1.29 ± 0.01	8.50 ± 0.19	1.70 ± 0.08	8.44 ± 0.17
Ethanol	0.00 ± 0.00	1.48 ± 0.29	0.13 ± 0.06	2.55 ± 0.26	0.25 ± 0.04	2.73 ± 0.11	0.28 ± 0.01	2.93 ± 0.17
	0.00 ± 0.00	1.54 ± 0.04	0.32 ± 0.06	2.95 ± 0.10	0.50 ± 0.21	2.03 ± 0.22	0.50 ± 0.01	4.03 ± 0.21
70% Ethanol methanol	0.18 ± .00	6.40 ± 0.84	0.62 ± 0.08	6.19 ± 0.32	0.78 ± 0.11	5.92 ± 0.04	0.81 ± 0.01	5.12 ± 0.06
	1.36 ± 0.09	6.06 ± 0.61	1.55 ± 0.05	8.51 ± 0.66	1.52± 0.04	6.67 ± 0.55	1.57 ± 0.10	5.57 ± 0.20
Methanol	0.00 ± 0.00	3.73 ± 0.48	0.15 ± 0.01	4.33 ± 0.16	0.20 ± 0.06	4.40 ± 0.47	0.44 ± 0.07	4.36 ± 0.20
	0.17 ± 0.08	5.35 ± 0.09	0.71 ± 0.07	6.41 ± 0.23	0.72 ± 0.19	6.45 ± 0.39	1.20 ± 0.14	6.67 ± 0.22
70% Methanol	0.22 ± 0.01	5.74 ± 0.08	0.68 ± 0.08	5.92 ± 0.07	0.68 ± 0.09	5.68 ± 0.076	0.80 ± 0.06	5.35 ± 0.50
	1.51 ± 0.14	8.11 ± 0.43	1.81 ± 0.12	8.91 ± 0.51	1.58 ± 0.02	8.41 ± 0.15	1.50 ± 0.06	8.34 ± 0.75
Acetone	0.00 ± 0.00	1.07 ± 0.02	0.21 ± 0.06	1.12 ± 0.05	0.30 ± 0.01	1.19 ± 0.38	0.50 ± 0.04	1.54 ± 0.09
	0.00 ± 0.00	1.36 ± 0.14	0.29 ± 0.21	1.23 ± 0.19	0.45 ± 0.12	1.43 ± 0.27	0.55 ± 0.06	2.27 ± 0.1
70% Acetone	0.40 ± 0.00	5.03 ± 0.22	0.70 ± 0.01	4.83 ± 0.24	0.97 ± 0.04	5.88 ± 0.01	1.10 ± 0.06	5.89 ± 0.26
	1.67 ± 0.01	4.85 ± 0.75	1.75 ± 0.01	4.99 ± 0.30	1.80 ± 0.05	6.05 ± 0.23	2.02 ± 0.05	6.28 ± 0.46

Various mechanisms have been identified for the antioxidant property of different plant extracts such as radical scavenging, binding of transition metal ion catalysts, decomposition of peroxides, prevention of chain reactions, prevention of continued hydrogen abstraction etc. About 20 assay methods are already available in literature for the estimation of antioxidant property [23]. DPPH scavenging assay is a widely used and one of the easiest method to evaluate the antioxidant property of a sample within a very short time period. DPPH is a stable free radical with purple color. Through electron transfer or hydrogen atoms donation, antioxidant compounds neutralize the free radical character of DPPH and thus purple color of the reaction mixture is changed to yellow [2].

Table 1 shows the DPPH scavenging property of unfermented and fermented wheat, extracted at different temperatures with different solvents. Increasing the extraction temperature from 30 °C to 60 °C, TPC as well as antioxidant activity were increased in unfermented wheat. Similar to TPC, maximum DPPH scavenging property (2.02 μmol TE g^−1^ grain) was observed in 70% acetone extract of unfermented wheat at 60 °C. Similarly, Zhou et al. [20] showed 50% acetone as a better solvent as compared to 50% methanol, for the extraction of antioxidant compounds from wheat.

Whereas, in case of fermented wheat, maximum %DPPH scavenging property was attained at 40 °C with equivalent amount of scavenging activity in water (8.85 μmol TE g^−1^ grain), 70% ethanol (8.51 μmol TE g^−1^ grain) and 70% methanol (8.91 μmol TE g^−1^ grain). Therefore, 40 °C was selected as the optimum temperature for the extraction of antioxidant compounds from *R. oryzae* fermented wheat. It was also observed that DPPH scavenging property of water soluble phenolics extract of wheat was increased by ∼7 fold (at 40 °C) in fermented wheat as compared to unfermented sample. Therefore, it has been proved that SSF is a fruitful method for the extraction/production of phenolics antioxidants from wheat. *Cordyceps militaris* was used by Zhang et al. [32] for the production of antioxidant supplements via SSF of wheat, however, with very less amount of improvement in antioxidant properties and 70% acetone was proved as the best extraction solvent. Comparisons in antioxidant properties of cereals results among individual research laboratories and groups are very difficult because different solvent systems and extracting conditions have been employed [20]. As fermented wheat was proved as a better source of antioxidant phenolic components, it was used for the further study and two other extraction conditions were optimized.

#### 3.1.2 Effect of solid-to-solvent ratio

**Fig. 1:**
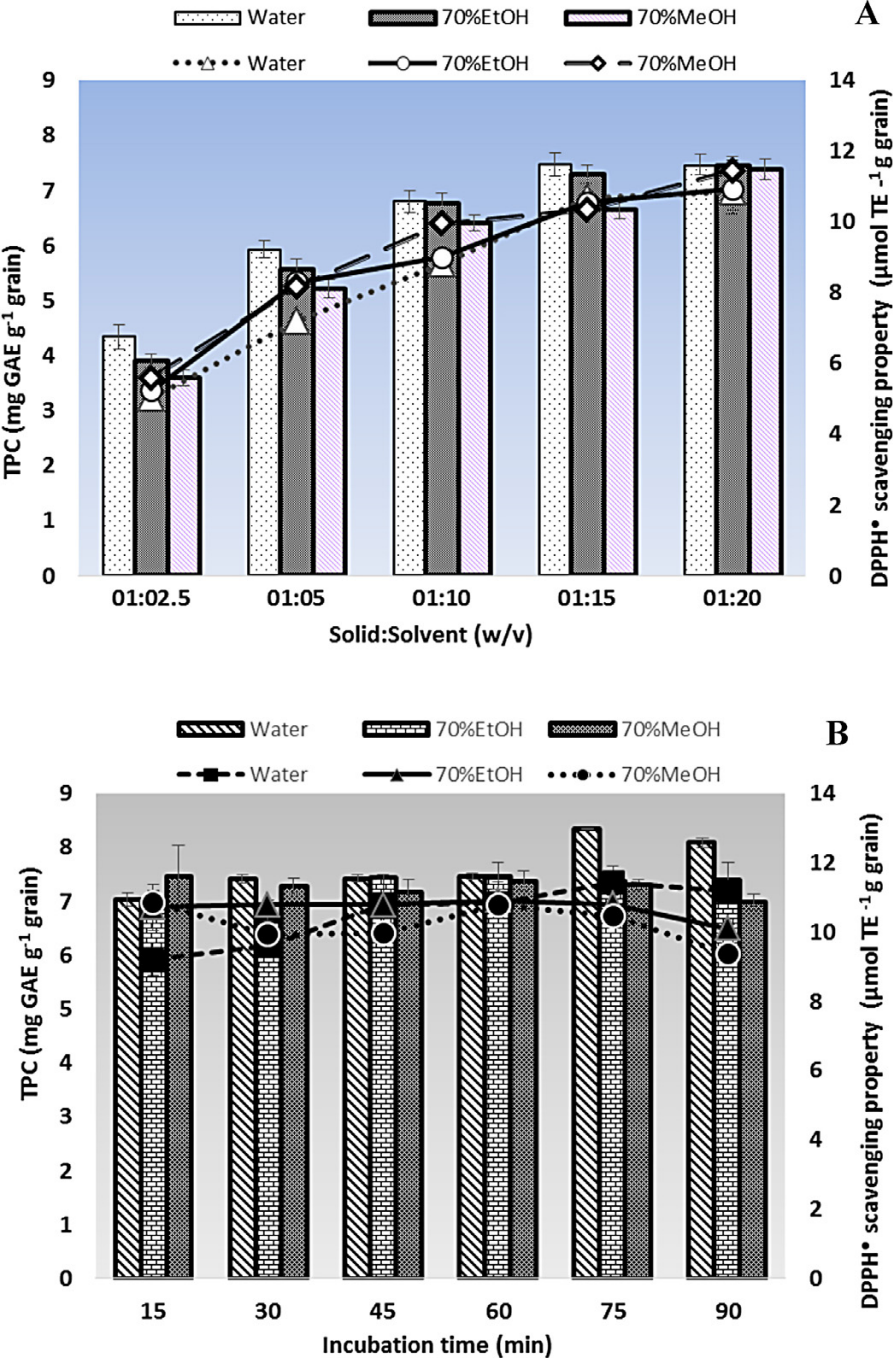
Effect of solid-to-solvent ratio (A) and extraction time (B) for the extraction of antioxidant phenolic compounds.

Solid-to-solvent ratio showed a significant effect for both TPC and DPPH scavenging property as shown in Fig. 1(A). Among all the ratios, solid-to-solvent at 1:15 (w/v) exhibited highest amount of DPPH scavenging property as well as TPC for water extract of *R. oryzae* fermented wheat. Zhang et al. [32] and Bhanja et al. [2] used solid-to-solvent ratio of 1:10 (w/v) for the extraction of phenolic antioxidants from fermented wheat. To our knowledge, there is no report available on the optimization of solid-to-solvent ratio for the extraction of phenolics from wheat or wheat based sample.

#### 3.1.3 Effect of extraction time

Extraction time is crucial in minimizing energy and cost of the extraction process. Effect of extraction time is shown in Fig. 1(B). Extraction time of 75 min was chosen as the optimum extraction time with maximum TPC of 8.33 mg GAE g^−1^ grain and DPPH scavenging property of 11.4 μmol TE g^−1^ grain. Liyana-Pathirana and Shahidi [8] optimized the conditions of phenolics extraction from whole grain wheat through response surface methodology (RSM) and found that the optimal condition for the total antioxidant activity of wheat was 54% ethanol as solvent, 61 °C as extraction temperature and 64 min as extraction time. In the present study, water was selected for the most suitable extracting solvent because it is the cheapest solvent and water extract of cereal grain fractions are of greatest relevance to *in vivo* activity as they contain water soluble antioxidants and thus more bioaccessible from food matrix in the digestive tract [13].

### 3.2 Comparative study of UFW and ROFW freeze-dried extracts

#### 3.2.1 Extraction yields, TPC and IC_50_ of DPPH and ABTS^•+^ scavenging property and *in vivo* antioxidant capacity

The extraction yields and TPC of water extracts are shown in Table 2. Significantly higher (*p* < 0.05) extraction yield was found with ROFW [25.88%] than the UFW [6.07%]. Extraction yield was observed to be increased after SSF, which was mainly due to the fact that after colonization of fungus, wheat was degraded and more water soluble substances like phenolics, sugars, organic acids and pigments were released [3]. Same concentration (10 mg/ml) of each freeze-dried extract was prepared in water and TPC was compared. TPC of the fermented wheat extract was significantly higher (*p* < 0.05) than UFW due to the release of more water soluble PCs by SSF.

**Table 2:** Extraction yields, TPC and IC_50_ of DPPH and ABTS^•+^ scavenging property and *in vivo* antioxidant activity of UFW and ROFW.

Sample	Extraction yield % (w/w)	Total phenolics content (mg GAE g^−1^ extract)	IC_50_ of DPPH• (mg/ml)	IC_50_ of ABTS^+^ (mg/ml)	% yeast cell growth
UFW	6.07 ± 0.70^a^	5.15 ± 0.22^a^	5.25 ± 0.053^a^	121.44 ± 8.65^a^	22.36 ± 1.2^a^
ROFW	25.88 ± 0.53^b^	24.55 ± 0.74^b^	0.64 ± 0.004^b^	34.93 ± 0.42^b^	31.55 ± 2.1^b^
Vit C	–	–	0.01 ± 0.001^c^	0.24 ± 0.01^c^	30.81 ± 2.8^b^

Values were expressed as means ± standard deviation. Values marked by the different lower-case superscript letters (from “a” to “c”) within a column denote statistically significant differences (*p* < 0.05).

The IC_50_ value was defined as the concentration of the sample required for 50% inhibition. The value was calculated by interpolation of linear regression analysis. IC_50_ values for DPPH scavenging property of UFW and ROFW were 5.25 and 0.64 in mg/ml, respectively (Table 2).

ABTS^•+^ scavenging assay is another method for the determination of free radical scavenging property of antioxidants. Reaction between ABTS and potassium persulfate produces blue colored ABTS^•+^ and decrease in the absorbance is caused by antioxidant phenolic compounds which reduce this preformed cation radical. In case of ABTS^•+^ scavenging property, the IC_50_ values of UFW and ROFW were 121.44 mg/ml and 34.93 mg/ml, respectively (Table 2). The lower IC_50_ values of ROFW in both the cases presented relatively higher radical scavenging activity.

Antioxidant properties of UFW and ROFW estimated i*n vivo* using *S. cerevisiae* are presented in Table 2. ROFW showed strong activity against H_2_O_2_, which was comparable with Vit C in same concentration (10 mg/ml). However, UFW showed less antioxidant activity against H_2_O_2_.

#### 3.2.2 FRAP, Hydroxyl radical and H_2_O_2_ scavenging property

**Fig. 2:**
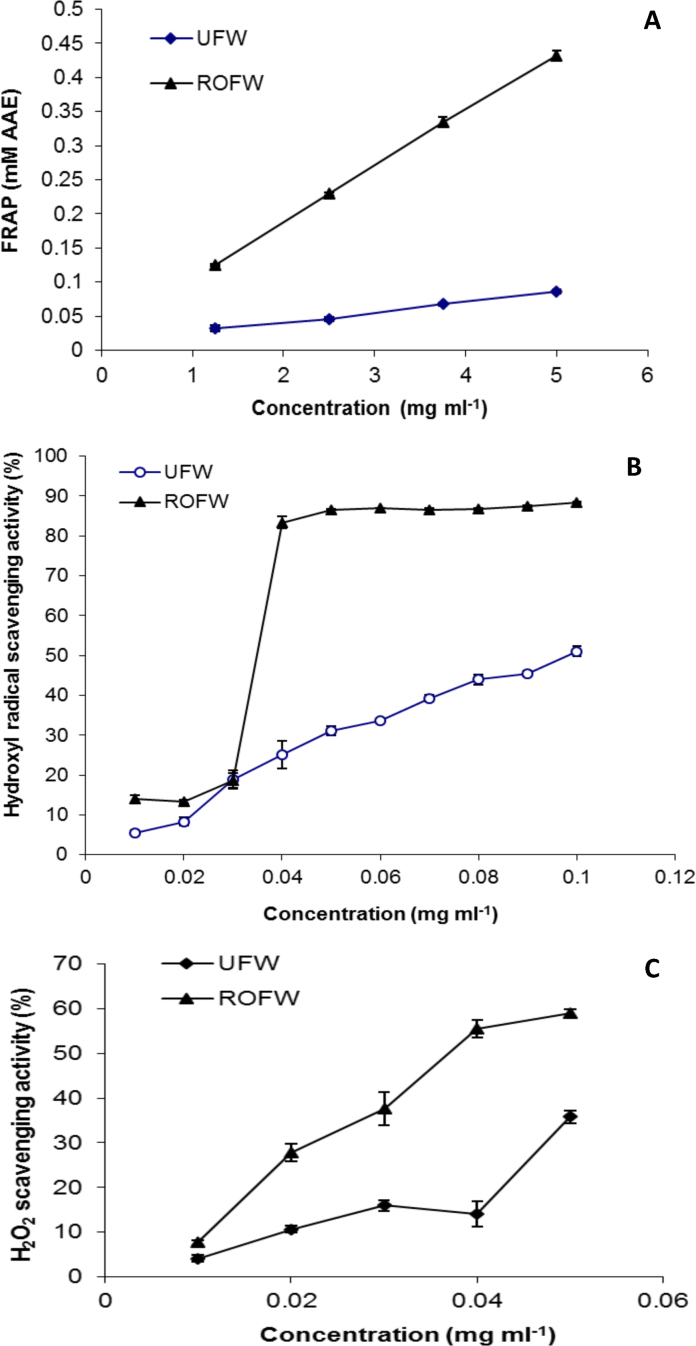
FRAP (A), hydroxyl radical (B) and H_2_O_2_ scavenging property (C) (UFW: unfermented wheat; ROFW: *Rhizopus oryzae* fermented wheat).

In FRAP assay system, antioxidant components reduce ferric–tripyridyltriazine complex to colored ferrous–tripyridyltriazine complex [3]. Fig. 2(A) shows the reducing power of UFW and ROFW extracts. ROFW showed higher FRAP at each concentration. The reducing property of tested samples indicates that they are electron donors. This result shows that SSF can improve the ferric reducing power of the wheat.

Hydroxyl radicals (OH) generated during the very well-known Fenton reaction degrade DNA deoxyribose with the help of Fe^2+^ as an important catalyst and may cause DNA strand breakage or DNA fragmentation [14]. The inhibition of OH mediated deoxyribose damage was determined by hydroxyl radical scavenging assay. As shown in Fig. 2(B), the water extract of ROFW exhibited dose-dependence (0.01–0.1 mg/ml) of hydroxyl radical scavenging activity. The scavenging effect of fermented wheat extract was higher than that of UFW at all the concentrations tested. In this assay, the IC_50_ values of UFW and ROFW were 0.093 mg/ml and 0.04 mg/ml, respectively. ROFW extract had lowest value of IC_50_ showing the maximum hydroxyl radical scavenging property.

H_2_O_2_ itself is not an extremely reactive oxygen species but it may give rise to OH which is a very toxic to cell. In the present study, all the samples were capable of scavenging H_2_O_2_ in a dose-dependent manner (Fig. 2(C)). The H_2_O_2_ scavenging effect of same dose (0.05 mg/ml) of water extracts decreased in the order of ROFW [59.0%]* > *UFW [35.8%]. The IC_50_ values of UFW and ROFW were 0.08 mg/ml and 0.04 mg/ml, respectively. The lowest IC_50_ value of ROFW represents maximum H_2_O_2_ scavenging property.

### 3.3 Profile of phenolic compounds

**Fig. 3:**
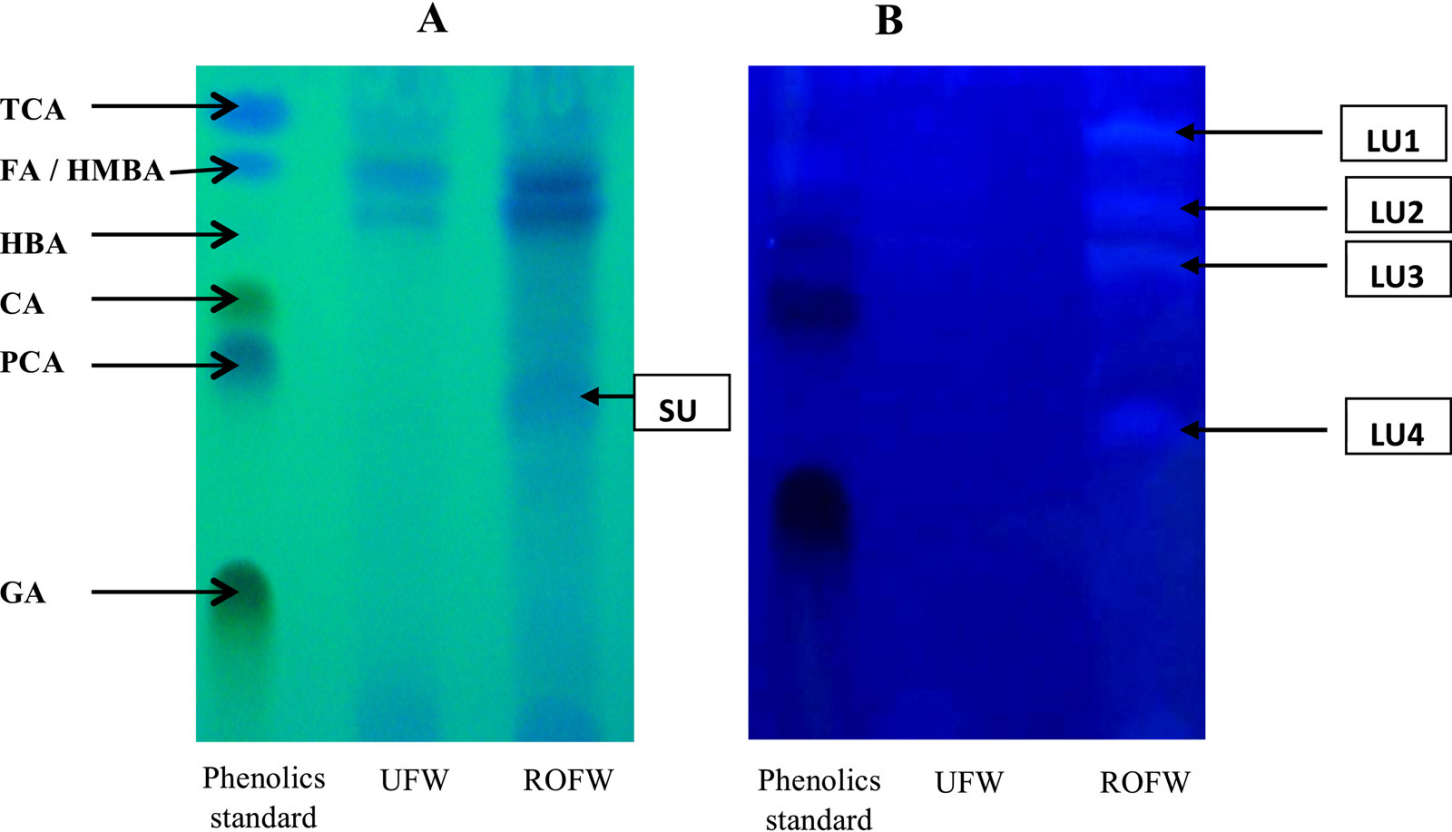
TLC profile of UFW and ROFW when water extract was phase separated by ethyl acetate, dried and dissolved in methanol recorded under short wave UV (A) and long wave UV (B) [GA: gallic acid; PCA: protocatecheuic acid; HBA: 4-hydroxybenzoic acid; HMBA: 4-hydroxy-3-methoxybenzoic acid; CA: caffeic acid; FA: ferulic acid; TCA: trans-cinnamic acid; SU: unknown compound in short wave UV; LU: unknown compound in long wave UV].

**Fig. 4:**
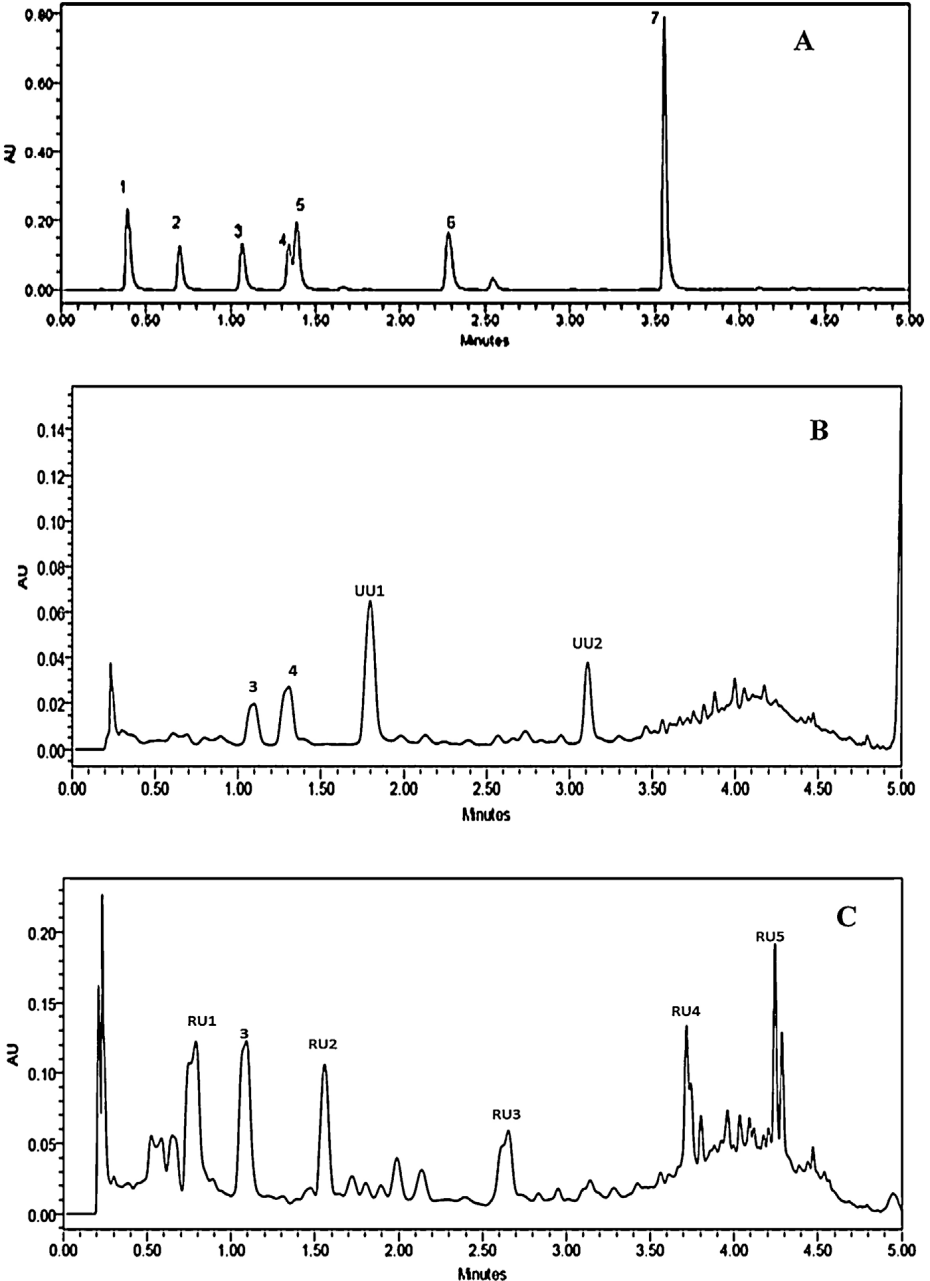
UPLC profile of standard phenolic compounds (A), UFW (B) and ROFW (C) at 280 nm [1: gallic acid; 2: protocatecheuic acid; 3: 4-hydroxybenzoic acid; 4: 4-hydroxy-3-methoxybenzoic acid; 5: caffeic acid; 6: ferulic acid; 7: trans-cinnamic acid; UU: unknown compound in UFW; RU: unknown compound in ROFW].

TLC and UPLC profiles of phenolics extracted from unfermented and *R. oryzae* RCK2012 fermented wheat are shown in Fig. 3, Fig. 4, respectively. Seven standards were separated within 5 min in UPLC. In case of UFW, the main phenolic compounds detected in UPLC as well as in TLC short wave UV were 4-hydroxy benzoic acid (HBA; 0.26 mg/g wheat) and 4-hydroxy-3-methoxybenzoic acid (HMBA; 0.22 mg/g wheat), whereas, two other unknown compounds (UU1 and UU2) were detected in UPLC. According to TLC of ROFW sample, three bands were identified in short wave UV namely HBA, HMBA and an unknown compound (SU), while, in long wave UV four unknown bands were observed (LU1-4). In UPLC profile of ROFW, HBA (1.61 mg/g wheat) and different unknown phenolic compounds (majorly RU1-5) were detected. Those unknown compounds might be contributing big role for the antioxidant property of ROFW. Through UPLC analysis [17], observed that syringic acid was the main compound (75.3–77%) in the free phenolic extracts (80% ethanol) of wheat meal. In our study, water was used as extracting solvent. Water extracts were phase separated by ethyl acetate, dried, dissolved in methanol and then they were injected in UPLC column. It is very difficult to compare our data with literature because different methods have been used for extraction. Moreover, antioxidant property varies between species and varieties of grains [34].

SSF is a complex biochemical process where several hydrolyzing enzymes like α-amylase, xylanase, β-glucosidase, esterases, etc., are produced, which are predicted to be associated in the release of water soluble and more bioavailable PC from insoluble bound form [4]. Along with enzymatic release of PC, some other unknown biochemical pathways might be involved in SSF process to increase the TPC and antioxidant properties of wheat. Moreover, in addition to PC, some other water soluble bioactive compounds like small peptides, xylo-oligosaccharides etc., produced during SSF might be contributing in the enhancement of antioxidant properties of fermented wheat [19], [9], [28].

## 4 Conclusion

Present study demonstrates that SSF of wheat by *R. oryzae* RCK2012 is a very fruitful method for the enhancement of TPC and antioxidant potential. On the basis of the results obtained, it is clearly indicated that water extract of ROFW has strong antioxidant property against several *in vitro* and an *in vivo* oxidative system compared to unfermented wheat. Predominantly insoluble bound phenolics in cereals are less bioavailable. To maximize the possible health benefits of cereals, SSF is a great option for the improvement of bioavailability of cereal phenolics by increasing their solubility. In comparison to unfermented wheat, consumption of fermented wheat might give more health protection against oxidative damages. Moreover, fermented extract can be served as powerful sources of natural antioxidants over the synthetic antioxidant compounds used very often in food and pharmaceutical industry. Additionally, along with PCs some other bioactive compounds might be produced during SSF, which were contributing antioxidant property. SSF process could be an innovative technology in cereal science research to develop nutrition rich and more healthy cereal products. Fermented wheat extract contains a complex mixture of phenolics. Further study is necessary to identify the unknown phenolic compounds.
